# Determinants of Gastroesophageal Reflux Disease, Including Hookah Smoking and Opium Use– A Cross-Sectional Analysis of 50,000 Individuals

**DOI:** 10.1371/journal.pone.0089256

**Published:** 2014-02-21

**Authors:** Farhad Islami, Siavosh Nasseri-Moghaddam, Akram Pourshams, Hossein Poustchi, Shahryar Semnani, Farin Kamangar, Arash Etemadi, Shahin Merat, Masoud Khoshnia, Sanford M. Dawsey, Paul D. Pharoah, Paul Brennan, Christian C. Abnet, Paolo Boffetta, Reza Malekzadeh

**Affiliations:** 1 Digestive Oncology Research Center, Digestive Disease Research Institute, Tehran University of Medical Sciences, Tehran, Iran; 2 The Tisch Cancer Institute and Institute for Transitional Epidemiology, Mount Sinai School of Medicine, New York, New York, United States of America; 3 Golestan Research Center of Gastroenterology and Hepatology, Golestan University of Medical Sciences, Gorgan, Iran; 4 Department of Public Health Analysis, School of Community Health and Policy, Morgan State University, Baltimore, Maryland, United States of America; 5 Division of Cancer Epidemiology and Genetics, National Cancer Institute, National Institutes of Health, Bethesda, Maryland, United States of America; 6 Departments of Oncology and Public Health and Primary Care, University of Cambridge, Cambridge, United Kingdom; 7 International Agency for Research on Cancer, Lyon, France; 8 International Prevention Research Institute, Lyon, France; University Hospital Llandough, United Kingdom

## Abstract

**Background:**

Gastroesophageal reflux disease (GERD) is a common cause of discomfort and morbidity worldwide. However, information on determinants of GERD from large-scale studies in low- to medium-income countries is limited. We investigated the factors associated with different measures of GERD symptoms, including frequency, patient-perceived severity, and onset time.

**Methods:**

We performed a cross-sectional analysis of the baseline data from a population-based cohort study of ∼50,000 individuals in in Golestan Province, Iran. GERD symptoms in this study included regurgitation and/or heartburn.

**Results:**

Approximately 20% of participants reported at least weekly symptoms. Daily symptoms were less commonly reported by men, those of Turkmen ethnicity, and nass chewers. On the other hand, age, body mass index, alcohol drinking, cigarette smoking, opium use, lower socioeconomic status, and lower physical activity were associated with daily symptoms. Most of these factors showed similar associations with severe symptoms. Women with higher BMI and waist to hip ratio were more likely to report frequent and severe GERD symptoms. Hookah smoking (OR 1.34, 95% CI 1.02–1.75) and opium use (OR 1.70, 95% CI 1.55–1.87) were associated with severe symptoms, whereas nass chewing had an inverse association (OR 0.87, 95% CI 0.76–0.99). After exclusion of cigarette smokers, hookah smoking was still positively associated and nass chewing was inversely associated with GERD symptoms (all frequencies combined).

**Conclusion:**

GERD is common in this population. The associations of hookah and opium use and inverse association of nass use with GERD symptoms are reported for the first time. Further studies are required to investigate the nature of these associations. Other determinants of GERD were mostly comparable to those reported elsewhere.

## Introduction

Gastroesophageal reflux disease (GERD) has increased in Europe and the United States over the past decades [1]–[3]. GERD symptoms are among the most common gastrointestinal symptoms in those regions [4], with prevalence rates of 10–25% reported from population-based studies [2], [5]–[8]. Several population-based studies from Iran, in West Asia, have reported prevalence rates similar to those in Western countries [9]–[11]. The incidence of GERD is increasing in Iran [12], and currently it is the most common outpatient gastrointestinal disease encountered there [13].

Determinants of GERD in the general population have been examined in a number of studies [14]–[24], but some potential determinants have shown conflicting results and are yet to be established. Also, data from low- to medium income countries are limited, as only a few of the population-based studies on determinants of GERD have been conducted in those countries [25]–[30]. We aimed to investigate determinants of prevalent GERD with cross-sectional analysis of the baseline data from the Golestan Cohort Study, a prospective cohort of over 50,000 individuals in Golestan Province, in northeastern Iran. We analyzed the data on frequency, patient-perceived severity, and the time of the first episode of GERD symptoms.

## Methods

### Study Population

The Golestan Cohort Study was primarily designed to investigate risk factors for upper gastrointestinal cancers. The design of this cohort has been described elsewhere [31]. Briefly, the Golestan Cohort Study is a prospective population-based cohort of 40–75 years old individuals in eastern parts of Golestan Province, Iran. Urban inhabitants in the specified age range were selected randomly from Gonbad City, the main urban area in eastern Golestan, by systematic clustering based on the household number. In rural areas, all residents of 326 villages in the study catchment area in the specified age range were invited to participate. A total of 50,045 adults without history of upper gastrointestinal cancers were enrolled in the study between January 2004 and June 2008.

### Ethics Statement

Written consent was obtained from all participants. The conduct of the Golestan Cohort Study, including the consent procedure, was approved by the Institutional Review Boards of the Digestive Disease Research Center of Tehran University of Medical Sciences, the US National Cancer Institute, and the International Agency for Research on Cancer.

### Exposure Measurements

At baseline, trained nurses and physicians conducted face-to-face interviews using structured questionnaires to collect data on GERD, potential determinants of GERD, and confounding factors. Weight, height, and waist and hip circumferences were measured by trained research staff. Body mass index (BMI) was calculated by dividing weight (kg) by the squared value of height (m).

**Figure 1:**
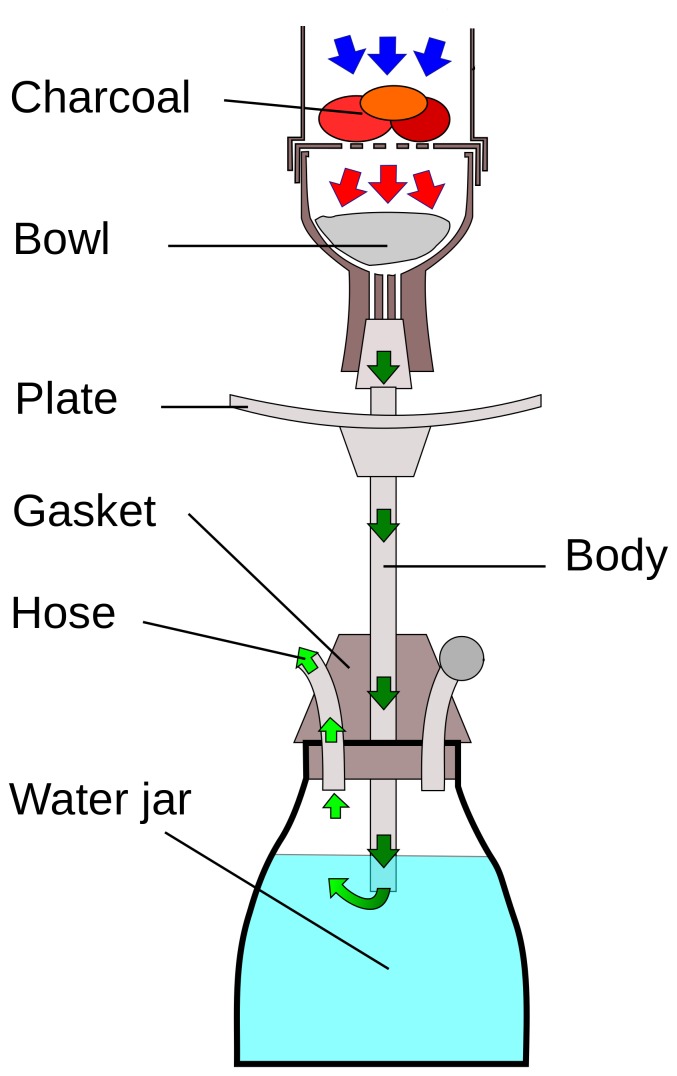
Diagram of a hookah. Source: Wikipedia (http://en.wikipedia.org/wiki/File:Hookah-lookthrough.svg), after modification.

Individuals who had ever used alcohol, cigarettes, hookah (also known as water-pipe, shisha, nargileh, and qalyan), nass (a mixture of tobacco, lime, and ash), or opium at least once a week for a period of 6 months or more were considered as users of the respective substance. In hookah smoking, tobacco is placed at the top of the hookah inside a bowl, which is separated with a perforated metal foil from burning coal placed on top [32]. Hookah smoke passes through a water basin and cools down, and then it is inhaled using a hose attached to the upper part of the water basin (Figure 1). Some people may believe that hookah smoking is harmless, assuming that its harmful compounds are filtered in the water [32]. However, there are several biomarker studies in humans that have shown appreciable amounts of tobacco related-compounds following hookah smoking [33]–[35], refuting the harmlessness of hookah. Although cigarette and hookah are both tobacco smoking produzcts, we considered them as separate entities because patterns of use of these products might be different, and there have been few published studies on the association between hookah smoking and GERD. We calculated cumulative amount of cigarette use (as pack-years) using data on duration and quantity of use. In accord with our earlier publications [36], we calculated a composite score for wealth by applying multiple correspondence analysis to appliance ownership data (including personal car, motorbike, black and white TV, color TV, refrigerator, freezer, vacuum cleaner, and washing machine). We only considered occupational physical activity because recreational physical activity is uncommon in the study population.

### Outcome Measurements

We asked the study participants about having regurgitation or heartburn over the past year and prior to the past year. Those with any either the symptoms in either time period were considered as having GERD symptoms. The frequency of GERD symptoms was recorded as never, occasional (including those associated with certain foods or drinks only), 1–3 times/month, once a week, 2–6 times/week, and daily. We combined the frequencies as never, <weekly (combination of occasional and 1–3 times/month), weekly (combination of once a week and 2–6 times/week), and daily for our analyses. We also asked about the severity of symptoms, which were categorized as: “mild”, the study participant did not feel the symptoms unless they actively paid attention; “moderate”, the study participant felt the symptom anyway, but it did not interfere with daily work; and “severe”, symptoms interfering with daily work or causing night-time awakenings. The frequency and severity of GERD symptoms were asked separately for the past year and for prior to one year before the interview. As the reported frequencies and severities for these two periods were comparable (Table S1) and we had another variable on the starting time of the symptoms, we combined the data and considered the most frequent frequency and the most severe severity of GERD symptoms in either of the two periods as the usual frequency and severity of symptoms, respectively. The first episode of GERD was recorded as within the last year, and 1–5, 6–10, or >10 years ago.

### Statistical Analysis

The number of individuals with missing values in all GERD variables (<0.1% of the cohort participants) and in individual GERD variables (<0.7% for each of the variables) was small, so the first group was excluded from the current analyses, and the second group was excluded from the analyses of the respective variable. Numbers and percentages were calculated and presented for categorical variables, as well as means and standard deviations for continuous variables. Odds ratios (ORs) and 95% confidence intervals (95% CIs) for the association of sociodemographic and lifestyle factors and anthropometric indices with frequency and severity of GERD symptoms were calculated using multinomial logistic regression models. In the analyses of frequency, <weekly, weekly, and daily symptoms, and in the analyses of severity, mild, moderate, and severe symptoms, as separate categories were compared with never having GERD symptoms. *P* values for trend were obtained from the same multinomial logistic regression models by assigning consecutive numbers to categories within each categorical variable.

Multivariate models were adjusted for several potential confounding factors as indicated in the table footnotes. As participants in our study could have shifted from using cigarettes to hookah or nass following the development of GERD, we also investigated the associations between hookah and nass use and GERD among never-cigarette smokers. All statistical analyses were performed using Stata statistical software version 11 (Stata Corporation, College Station, Texas, USA). All reported *P* values are two-sided, and *P*<0.05 was considered to be statistically significant.

## Results

Data on reflux were available for 50,001 individuals. Approximately 12% of participants reported daily and 11% reported severe GERD symptoms; 16% of participants reported GERD symptoms with the first episode happening >10 years before the interview (Table 1).

**Table 1:** GERD symptoms in 50,001 individuals with data on GERD in the Golestan Cohort Study.

GERD symptoms	Number (%)
**Symptom frequency**	
Never	19,560 (39.12)
<Weekly	20,471 (40.94)
Weekly	4029 (8.06)
Daily	5915 (11.83)
Missing	26 (0.05)
**Symptom severity**	
Mild	4449 (8.90)
Moderate	20,315 (40.63)
Severe	5663 (11.33)
Missing	16 (0.03)
**Symptom start**	
<1 year ago	5326 (10.65)
1–5 years ago	12,534 (25.07)
6–10 years ago	4444 (8.89)
>10 years ago	7895 (15.79)
Missing	304 (0.61)

GERD, gastroesophageal reflux disease.

Daily GERD symptoms had inverse associations with being a male (OR 0.36, 95% CI 0.33–0.39) or of Turkmen ethnicity (OR 0.66, 95% CI 0.61–0.70), formal education (*P*_trend_ 0.01), wealth score (*P*_trend_ <0.001), regular non-intense physical activity (OR 0.90, 95% CI 0.83–0.98), and nass chewing (OR 0.86, 95% CI 0.75–0.98) (Table 2). On the other hand, daily symptoms were positively associated with older age (7% increase in risk per 10-year increase in age), higher BMI (*P*_trend_ <0.001), alcohol drinking (OR 1.36, 95% CI 1.13–1.64), cigarette smoking (OR 1.43, 95% CI 1.23–1.67 for smoking ≥20 pack-years), and opium use (OR 1.82, 95% CI 1.67–1.99). The association between age and daily symptoms was linear (data not shown).

**Table 2:** Association of several demographic and lifestyle factors with daily and severe GERD symptoms.

Variables	All	No symptoms	Daily symptoms		Severe symptoms	
	N (%)	N (%)	N (%)	OR (95% CI)	N (%)	OR (95% CI)
**Total**	50,001 (100)	19,560 (100)	5915 (100)	–	5663 (100)	–
**Age** *	52.1 (9.0)	52.1 (9.0)	52.7 (9.2)	1.07 (1.04–1.11)	52.2 (8.9)	1.01 (0.98–1.05)
**Sex**						
Women	28,785 (57.57)	9947 (50.85)	4241 (71.70)	Referent	3981 (70.30)	Referent
Men	21,216 (42.43)	9613 (49.15)	1674 (28.30)	0.36 (0.33–0.39)	1682 (29.70)	0.36 (0.33–0.39)
**Ethnicity**						
Non-Turkmen	12,786 (25.57)	4913 (25.12)	2001 (33.83)	Referent	1477 (26.08)	Referent
Turkmen	37,215 (74.43)	14,647 (74.88)	3914 (66.17)	0.66 (0.61–0.70)	4186 (73.92)	0.98 (0.91–1.06)
**Residence**						
Rural	39,366 (78.73)	15,962 (81.61)	4802 (81.18)	Referent	4518 (79.78)	Referent
Urban	10,634 (21.27)	3598 (18.39)	1113 (18.82)	1.01 (0.93–1.11)	1145 (20.22)	1.13 (1.04–1.23)
**Education**						
No school	35,089 (70.18)	13,319 (68.09)	4672 (78.99)	Referent	4444 (78.47)	Referent
1–8^th^ grade	10,698 (21.40)	4479 (22.90)	985 (16.65)	0.92 (0.84–1.00)	919 (16.23)	0.84 (0.77–0.93)
High School	3150 (6.30)	1342 (6.86)	197 (3.33)	0.78 (0.66–0.93)	220 (3.88)	0.73 (0.62–0.87)
Higher	1064 (2.13)	420 (2.15)	61 (1.03)	0.92 (0.69–1.23)	80 (1.41)	0.99 (0.76–1.29)
* P* for trend				0.01		0.001
**Wealth score**						
Quintile 1-lowest	13,455 (26.91)	5089 (26.02)	1948 (32.93)	Referent	1888 (33.34)	Referent
Quintile 2	8469 (16.94)	3394 (17.35)	976 (16.50)	0.86 (0.79–0.94)	936 (16.53)	0.80 (0.73–0.87)
Quintile 3	9790 (19.58)	3845 (19.66)	1180 (19.95)	0.90 (0.83–0.98)	1111 (19.62)	0.80 (0.73–0.87)
Quintile 4	8345 (16.69)	3344 (17.10)	933 (15.77)	0.82 (0.75–0.90)	801 (14.14)	0.66 (0.60–0.73)
Quintile 5	9942 (19.88)	3888 (19.88)	878 (14.84)	0.68 (0.61–0.75)	927 (16.37)	0.65 (0.59–0.72)
* P* for trend				<0.001		<0.001
**Body mass index**						
<18.5 kg/m^2^	2410 (4.82)	989 (5.06)	324 (5.48)	0.94 (0.81–1.07)	298 (5.26)	0.96 (0.83–1.11)
18.5–24.9	17,914 (35.83)	7452 (38.11)	2083 (35.23)	Referent	1953 (34.50)	Referent
25–29.9	16,958 (33.92)	6576 (33.63)	1945 (32.89)	1.11 (1.04–1.20)	1840 (32.50)	1.10 (1.02–1.18)
≥30	12,710 (25.42)	4539 (23.21)	1561 (26.40)	1.15 (1.06–1.25)	1570 (27.73)	1.21 (1.11–1.31)
* P* for trend				<0.001		<0.001
**Physical activity**						
Irregular non-intense	30,619 (61.44)	11,579 (59.36)	4235 (71.86)	Referent	3750 (66.37)	Referent
Regular non-intense	13,524 (27.14)	5411 (27.74)	1086 (18.43)	0.90 (0.83–0.98)	1416 (25.06)	1.28 (1.18–1.38)
Regular or irregular intense	5691 (11.42)	2518 12.91)	572 (9.71)	0.94 (0.84–1.05)	484 (8.57)	0.97 (0.87–1.09)
* P* for trend				0.04		0.04
**Alcohol drinking**						
Never	48,274 (96.55)	18917 (96.71)	5740 (97.04)	Referent	5460 (96.42)	Referent
Ever	1727 (3.45)	643 (3.29)	175 (2.96)	1.36 (1.13–1.64)	203 (3.58)	1.53 (1.28–1.83)
**Cigarette smoking**						
Never	41,409 (82.84)	16,186 (82.77)	5066 (85.65)	Referent	4804 (84.83)	Referent
0.1–5 pack-years	2764 (5.53)	1118 (5.72)	268 (4.53)	1.20 (1.04–1.40)	266 (4.70)	1.24 (1.07–1.45)
5.1–10	1261 (2.52)	490 (2.51)	122 (2.06)	1.37 (1.10–1.70)	126 (2.22)	1.42 (1.15–1.76)
10.1–20	1799 (3.60)	692 (3.54)	154 (2.60)	1.21 (1.00–1.46)	177 (3.13)	1.40 (1.16–1.68)
≥20	2753 (5.51)	1069 (5.47)	305 (5.16)	1.43 (1.23–1.67)	290 (5.12)	1.42 (1.22–1.66)
* P* for trend				<0.001		<0.001
**Hookah smoking**						
Never	49,445 (98.93)	19,379 (99.12)	5812 (98.31)	Referent	5578 (98.55)	Referent
Ever	533 (1.07)	173 (0.88)	100 (1.69)	1.19 (0.92–1.54)	82 (1.45)	1.34 (1.02–1.75)
**Nass chewing**						
Never	46,224 (92.45)	17,989 (91.97)	5529 (93.47)	Referent	5300 (93.59)	Referent
Ever	3773 (7.55)	1570 (8.03)	386 (6.53)	0.86 (0.75–0.98)	363 (6.41)	0.87 (0.76–0.99)
**Opium use**						
Never	41,507 (83.02)	16,540 (84.56)	4738 (80.13)	Referent	4573 (80.78)	Referent
Ever	8488 (16.98)	3020 (15.44)	1175 (19.87)	1.82 (1.67–1.99)	1088 (19.22)	1.70 (1.55–1.87)

CI, confidence interval; GERD, gastroesophageal reflux disease; OR, odds ratio.

Numbers may not add up to the total numbers due to missing data. The ORs (95% CIs) were calculated using multinomial logistic regression models. In the analyses of frequency, <weekly, weekly, and daily symptoms, and in the analyses of severity, mild, moderate, and severe symptoms, as separate categories were compared with never having GERD symptoms. Results for <weekly and weekly symptoms are shown in Table S2. Results for mild and moderate symptoms are shown in Table S3. The ORs (95% CIs) are from multivariate models in which all the variables shown in this table were included.

* For age, the values are mean (standard deviation) years. Age was included in the models as a continuous variable, but the ORs (95% CIs) are shown here on a 10-year scale.

Being a male, having formal education or higher wealth scores, and chewing nass were inversely associated with reporting severe symptoms (Table 2). On the other hand, severe symptoms were positively associated with BMI, alcohol drinking, or cigarette, hookah, or opium use. In never cigarette smokers (Table 3), hookah smoking was positively associated (OR 1.26, 95% CI 1.01–1.56) and nass chewing was inversely associated (OR 0.85, 95% CI 0.76–0.94) with GERD symptoms (any frequency or severity combined).

**Table 3:** Association of hookah and nass use with GERD symptoms in never cigarette smokers.

Variables	Any GERD symptom		Frequency						Severity					
			<Weekly		Weekly		Daily		Mild		Moderate		Severe	
	N (%)	OR (95% CI)	N (%)	OR (95% CI)	N (%)	OR (95% CI)	N (%)	OR (95% CI)	N (%)	OR (95% CI)	N (%)	OR (95% CI)	N (%)	OR (95% CI)
**Hookah smoking**														
Never	40,973 (98.99)	Referent	16,625 (99.11)	Referent	3300 (98.42)	Referent	4980 (98.34)	Referent	3562 (98.73)	Referent	16,605 (98.92)	Referent	4743 (98.77)	Referent
Ever	418 (1.01)	1.26(1.01–1.56)	150 (0.89)	1.22(0.96–1.55)	53 (1.58)	1.32(0.95–1.83)	84 (1.66)	1.22(0.92–1.63)	131 (0.81)	1.41(1.00–1.99)	182 (1.08)	1.25(0.99–1.57)	59 (1.23)	1.19(0.87–1.64)
**Nass chewing**														
Never	39,965 (95.77)	Referent	16,198 (96.52)	Referent	3202(95.44)	Referent	4873 (96.19)	Referent	3468 (96.12)	Referent	16,183 (96.35)	Referent	4626 (96.29)	Referent
Ever	1751(4.23)	0.85(0.76–0.94)	584 (3.48)	0.84 (0.74–0.94)	153 (4.56)	1.01(0.83–1.23)	193 (3.81)	0.79(0.66–0.95)	140 (3.88)	1.02(0.83–1.25)	613 (3.65)	0.82(0.73–0.93)	178 (3.71)	0.85(0.71–1.03)

Controls are those with no heartburn or regurgitation. Numbers may not add up to the total numbers due to missing data. The odds ratios (95% confidence intervals) were adjusted for age, sex, ethnicity, place of residence, education, wealth score, body mass index, physical activity, and consumption of alcohol, opium, and the other tobacco product shown in this table (hookah or nass).

The associations with <weekly and weekly symptoms (Table S2) or mild to moderate symptoms (Table S3) in most cases were similar to those of daily or severe symptoms, respectively. However, those with education levels of above high school were more likely to report <weekly or mild to moderate symptoms than those with no formal education.

The associations of cigarette smoking and opium use and inverse association of nass use were stronger with longer duration of the time period between the onset of GERD symptoms and baseline interview (Table S4). As expected, age was also associated with this duration.

In women, both higher BMI and higher waist to hip ratio were associated with daily and severe symptoms (Table 4). The association between waist to hip ratio and reflux symptoms persisted after adjustments for BMI, suggesting an independent role of central obesity in GERD in women. Waist to hip ratio showed a trend of association with daily GERD symptoms in men (*P* for trend 0.04), but this association attenuated after adjustment for BMI. None of the categories of BMI or waist to hip ratio had statistically significant associations with GERD symptoms in men. The patterns of association between waist circumference and GERD symptoms in men and women were comparable with those of waist to hip ratio and GERD (data not shown).

**Table 4:** Association between anthropometric indices and daily and severe GERD symptoms by sex.

	All participants	No symptoms	Daily symptoms			Severe symptoms		
Variables	N (%)	N (%)	N (%)	OR 1 (95% CI)	OR 2 (95% CI)	N (%)	OR 1 (95% CI)	OR 2 (95% CI)
**Women**	28,785 (100)	9947 (100)	4241 (100)			3981 (100)		
**Body mass index**								
<18.5 kg/m^2^	1153 (4.01)	411 (4.13)	324 (5.48)	0.87 (0.72–1.05)	–	298 (5.26)	0.91 (0.75–1.11)	–
18.5–24.9	8311 (28.88)	3049 (30.66)	2083 (35.23)	Referent	–	1953 (34.50)	Referent	–
25.0–29.9	9691 (33.67)	3348 (33.66)	1945 (32.89)	1.12 (1.02–1.22)	–	1840 (32.50)	1.12 (1.02–1.23)	–
≥30	9627 (33.45)	3138 (31.55)	1561 (26.40)	1.21 (1.10–1.33)	–	1570 (27.73)	1.30 (1.18–1.43)	–
* P* for trend				<0.001	–		<0.001	
**Waist to hip ratio**								
***WHO Criteria***								
Normal	3091 (10.74)	1249 (12.56)	414 (9.77)	Referent	Referent	390 (9.81)	Referent	Referent
At risk (≥0.85)	25,680 (89.26)	8692 (87.44)	3824 (90.23)	1.34 (1.19–1.51)	1.25 (1.09–1.43)	3587 (90.19)	1.32 (1.17–1.49)	1.21 (1.05–1.38)
***Quintiles***								
<0.882	5551 (19.29)	2186 (21.99)	727 (17.15)	Referent	Referent	694 (17.45)	Referent	Referent
0.882–0.934	5772 (20.06)	1981 (19.93)	782 (18.45)	1.21 (1.07–1.36)	1.20 (1.06–1.36)	7376 (18.53)	1.18 (1.05–1.34)	1.15 (1.02–1.31)
0.935–0.978	5595 (19.45)	1919 (19.30)	824 (19.44)	1.33 (1.18–1.49)	1.31 (1.16–1.50)	813 (20.44)	1.34 (1.19–1.51)	1.29 (1.13–1.47)
0.979–1.027	5850 (20.33)	1909 (19.20)	904 (21.33)	1.43 (1.27–1.61)	1.42 (1.24–1.62)	1466 (20.64)	1.35 (1.20–1.53)	1.28 (1.12–1.47)
≥1.028	6003 (20.86)	1946 (19.58)	1001 (23.62)	1.52 (1.35–1.71)	1.50 (1.31–1.72)	1570 (22.93)	1.46 (1.29–1.65)	1.36 (1.18–1.57)
* P* for trend				<0.001	<0.001		<0.001	<0.001
**Men**	21216 (100)	9613 (100)	1674 (100)			1682 (100)		
**Body mass index**								
<18.5 kg/m^2^	1257 (5.93)	578 (6.01)	324 (5.48)	1.03 (0.84–1.27)	–	298 (5.26)	1.02 (0.82–1.26)	–
18.5–24.9	9603 (45.28)	4403 (45.82)	2083 (53.23)	Referent	–	1953 (34.50)	Referent	–
25.0–29.9	7267 (34.26)	3228 (33.59)	1945 (32.89)	1.11 (0.98–1.25)	–	1840 (32.50)	1.08 (0.96–1.22)	–
≥30	3083 (14.54)	1401 (14.58)	1561 (26.40)	1.06 (0.90–1.26)	–	1570 (27.73)	1.01 (0.85–1.19)	–
* P* for trend				0.17			0.42	
**Waist to hip ratio**								
***WHO Criteria***								
Normal	5457 (25.74)	2550 (26.55)	465 (27.79)	Referent	Referent	483 (28.75)	Referent	Referent
At risk (≥0.90)	15741 (74.26)	7054 (73.45)	1208 (72.21)	1.10 (0.97–1.24)	1.08 (0.93–1.24)	1197 (71.25)	1.05 (0.93–1.18)	1.03 (0.89–1.18)
***Quintiles***								
<0.883	4204 (19.83)	1980 (20.62)	361 (21.58)	Referent	Referent	375 (22.32)	Referent	Referent
0.883–0.929	4048 (19.10)	1789 (18.63)	324 (19.37)	1.04 (0.88–1.23)	1.05 (0.88–1.24)	321 (19.11)	1.01 (0.85–1.18)	1.00 (0.85–1.19)
0.930–0.971	4351 (20.53)	1954 (20.35)	324 (19.37)	1.05 (0.89–1.24)	1.05 (0.87–1.26)	339 (20.18)	1.06 (0.90–1.25)	1.05 (0.87–1.25)
0.972–1.018	4217 (19.89)	1868 (19.45)	321 (19.19)	1.14 (0.96–1.34)	1.13 (0.93–1.38)	332 (19.76)	1.13 (0.95–1.33)	1.11 (0.91–1.34)
≥1.019	4378 (20.65)	2013 (20.96)	343 (20.50)	1.15 (0.97–1.36)	1.15 (0.93–1.42)	313 (18.63)	1.02 (0.87–1.21)	1.01 (0.81–1.25)
* P* for trend				0.04	0.09		0.37	0.58

CI, confidence interval; GERD, gastroesophageal reflux disease; OR, odds ratio; WHO, World Health Organization.

Numbers may not add up to the total numbers due to missing data. The ORs (95% CIs) were calculated using multinomial logistic regression models. In the analyses of frequency, <weekly, weekly, and daily symptoms, and in the analyses of severity, mild, moderate, and severe symptoms, as separate categories were compared with never having GERD symptoms. Results for <weekly, weekly, mild, and moderate symptoms are not shown. OR 1s (95% CIs) were adjusted for age, sex, ethnicity, place of residence, education, wealth score, physical activity, consumption of alcohol, cigarette, hookah, nass, and opium (variables as shown in Table 2). OR 2s (95% CIs) were additionally adjusted for body mass index.

## Discussion

In this study, approximately 20% of participants had weekly or more frequent GERD symptoms. Several sociodemographic and lifestyle factors were associated with GERD symptoms. Many of these associations have been reported in other populations. We found an association between hookah or opium use and GERD symptoms and an inverse association between nass use and the symptoms for the first time. To the best of our knowledge, this is one of the largest studies on determinants of GERD symptoms worldwide and by far the largest study in low- and medium-income countries [37].

### Alcohol, Tobacco, and Opium Use

Associations of alcohol drinking and cigarette smoking with GERD symptoms and esophagitis have previously been reported (generally with OR <2), although these associations have not been shown in all studies [28], [38], [39]. We found modest but statistically significant associations between alcohol or cigarette use and GERD symptoms, with a significant exposure-response trend for the latter. Cigarette smoking usually starts in young adulthood, so temporal relationship between this habit and GERD is likely.

Ever hookah smoking was also associated with GERD. The association between hookah smoking and GERD (any symptoms) persisted even after exclusion of cigarette smokers. Among never cigarette smokers, hookah smoking had statistically significant or borderline significant associations with mild and moderate GERD symptoms, but the association with severe symptoms was non-significant. The number of hookah smokers with severe symptoms was modest, which may be a reason for the above pattern. The magnitude of association was slightly stronger with mild to moderate symptoms. This may be because many hookah smokers in our study smoked hookah recreationally and with relatively low frequencies, so the symptoms associated with hookah smoking might be more likely to be mild to moderate. In fact, approximately half of the hookah smokers in this study had smoked less than 11 unit-years, which was equivalent to smoking hookah only once a day for 11 years (data not shown). Due to the modest number of hookah smokers in our study, we were not able to investigate the association by categories of use. The association between hookah use and GERD symptoms may be explainable by the comparability of the exposures in cigarette and hookah smoking [40]. Cigarette smoking increases frequency of gastroesophageal reflux episodes by reducing the lower esophageal sphincter pressure [41] and reduces salivary secretion of bicarbonates [42]. However, some other mechanisms might also be involved in the association between hookah smoking and GERD symptooms. For example, mean puff volume in hookah smoking is generally over 500 mL [33], [43], [44], which is several times bigger than usual puff volumes in cigarette smoking (40–70 mL) [43]. Therefore, hookah smoking can induce strong negative intra-thoracic pressure and increase thoraco-abdominal pressure gradient, which may increase gastroesophageal reflux [45].

The reasons for the inverse association of nass use and positive association of opium use with GERD symptoms in our study are unclear. Nass contains tobacco specific *N*-nitroso compounds and volatile *N*-nitrosamines, but the levels of these compounds in nass seem to be lower than in chewing tobacco products in Western countries [46], [47]. Besides tobacco-related compounds, nass contains other compounds that are added during processing and have unknown effects on GERD symptoms. These ingredients increase the pH of nass to above 11 [46], whereas the pH of many other chewing tobacco products is between five to seven [46], [48]. The alkaline pH of nass may outweigh the potential harmful effects of tobacco with regard to GERD symptoms and may play a role in the inverse association between nass and the symptoms. Furthermore, using nass may be associated with increased saliva secretion and frequent swallowing, and similar to chewing gum [49], [50], it may reduce esophageal acid exposure. Morphine may reduce acid reflux in GERD patients [51], but the effects of morphine on GERD symptoms are unclear. Opium also contains several compounds other than morphine, including other opiate alkaloids (such as papaverine), non-alkaloid compounds from opium poppy (such as meconic acid), and other compounds added or generated during processing or smoking, including heterocyclic and polycyclic aromatic hydrocarbons and primary aromatic amines [52]–[55], which may have various, but yet unknown, effects on GERD symptoms. Opium is usually ingested or smoked [56].

The associations of hookah, nass, and opium use with GERD may all be true, but all of them were modest and may in part be related to the effects of unknown confounding factors or residual confounding. Furthermore, opium use might be secondary to the development of GERD symptoms, as some patients in this population may use opium for alleviation of their symptoms [56]. On the other hand, as hookah and cigarette smoke have several common compounds, a casual association between hookah smoking and GERD symptoms is plausible, assuming that cigarette smoking is causally associated with GERD. To the best of our knowledge, this is the first report on the association of hookah, nass, and opium use with GERD symptoms, and these associations merit further scrutiny. The investigations on hookah smoking may be of particular interest, as the prevalence of hookah smoking has been increasing among young adults in many populations, including in some European and North American countries [32].

### Sociodemographic Factors

Several, but not all [28], [38], [57], studies have reported a positive association between age and GERD, either as a linear association [26], [58] or with a peak and a slight decrease afterwards [59]–[61]. The histological damage in the esophageal epithelium, including esophagitis, may be more common in the elderly than in younger individuals [62]–[64], but older people may report severe symptoms less frequently [64]. In our study, age had a linear association with daily symptoms but was not associated with severe symptoms. Although the association between age and GERD appears to be causal, cohort effect may also play a role in the observed patterns of association, as the majority of the evidence comes from cross-sectional rather than longitudinal studies.

The current evidence on the association between gender and GERD symptoms is mixed, but the majority of studies have not shown any association [65]. However, in most studies with endoscopy, non-erosive GERD have been more common in women [66], whereas erosive esophagitis have been more common in men [65]–[67]. The reported prevalence of symptoms and histological damage related to GERD varies across ethnic/racial groups [68], [69]. We found a difference in prevalence of daily symptoms (but not in the severity of symptoms) between Turkmens and non-Turkmens. It is not clear what environmental and/or genetic factors contribute to these differences. The average perception of GERD symptoms may also vary in different socioeconomic and demographic groups, for example in ethnic/racial groups or in men and women [70]. At least part of the observed differences in the association of sociodemographic groups with GERD may be related to these differences in perception.

An association between poor socioeconomic status and GERD has been reported in other populations [18], [60], [71]. Our results also showed such an association even after adjustments for several other determinants of GERD. Reverse causality appears unlikely to explain the inverse association between education level and GERD, particularly for lower education levels, because education is usually started and completed at an early age, usually before the onset of GERD [60]. In our study, education levels of 1^st^–8^th^ grade and high school were the most commonly attained levels among those with formal education, and both showed inverse associations with daily and severe GERD symptoms. As socioeconomic status is not a biologic factor, the factors that are associated with socioeconomic status which may influence GERD symptoms need further investigations.

### Anthropometric Indices

The majority of previous studies have shown an association between higher BMI and GERD symptoms [72], [73]. Central adiposity seems to be a more important factor in this association than overall obesity [74]. The association between obesity and GERD seems to be causal, as exposure–response associations have been reported in multiple studies [72], [73], obesity has been associated with histological indicators of esophageal epithelium damage [72], [74], and weight loss has been associated with decreased GERD symptoms [15], [17].

Increased intra-abdominal pressure or thoraco-abdominal pressure gradients may be among the main possible explanations for the association of GERD with BMI and, in particular, central obesity [45], [75]. However, there seems to be other mechanisms contributing to this association, including reduced lower esophageal sphincter pressure in obese individuals [75], [76]. In any case, esophageal acid exposure has been positively associated with BMI [75] and waist circumference [76], [77]. The association between esophageal acid exposure and waist circumference has been reported in both groups of people with [76] or without [77] GERD symptoms.

In our study, high BMI and central obesity were associated with GERD symptoms in women. In men, central obesity showed trends for association with daily symptoms, but categories of neither BMI nor waist to hip ratio had significant associations with GERD symptoms. A stronger association between obesity and GERD symptoms or esophagitis in women [15], [70], [78], and an association between estrogen hormone therapy and GERD [15], [79] have previously been reported. However, several other studies have not shown a difference in the association between obesity and GERD by gender [68], [80]. The reasons for this variation in results are unclear. Some speculative explanations include: other risk factors for GERD may be so common in a population (or a subpopulation, such as men) that they may reduce the apparent effect of obesity. Also, we cannot exclude presence of unknown confounding factors or residual confounding. Furthermore, anthropometric indices may change after development of GERD. In this case, losing or gaining weight can reduce or increase, respectively, the association between obesity and GERD in cross-sectional studies.

### Physical Activity

Although vigorous exercise has been associated with GERD [81], moderate physical activity may have an inverse association with GERD symptoms in the general population [30], [82] or in obese patients only [83]. A study has also suggested a positive association between physical activity at work and GERD symptoms but an inverse association with recreational physical activity [84]. In our study, the associations between occupational physical activity and frequency or severity of GERD symptoms were mixed. These conflicting results may partly be related to variation in the definition and assessment of physical activity across studies. Also, a clinical trial has shown that actively training the diaphragm by breathing exercise may relieve GERD symptoms [85]. Therefore, different types of exercise and physical activity may have various effects on GERD depending on their impact on different parts of the body. Further longitudinal studies in this regard using standard measurement methods are required.

### Strengths and Limitations of the Study

A relatively large sample size, collection of detailed information on GERD symptoms and other factors, and adjustments for multiple potential confounding factors are among the strengths of this study. One limitation was the lack of data on endoscopic and histological damage associated with GERD. However, as GERD is a clinical diagnosis in most instances, especially in the primary care setting, and its symptoms are a common source of discomfort regardless of the presence or absence of endoscopic and histologic findings, investigation of determinants of GERD per se may have clinical implications. Another limitation was that we collected data only on regurgitation and heartburn and not on less common symptoms of GERD. However, the common definition of GERD is based on regurgitation and heartburn, and most studies have used this definition. Furthermore, using other less specific symptoms might have introduced substantial measurement error. For example, GERD can cause cough [38], but cough can also be related to many other disorders [86].

Cross-sectional studies may not be able to ascertain the temporal relationship between exposures and outcomes. However, this may not be a major drawback for some socio-demographic factors, including age, sex, ethnicity, and education. On the other hand, we did not analyze the collected dietary data (which covered dietary intakes over the one year before the interview) because of the probability of a modification in diet following GERD symptoms. However, we adjusted the results for several factors that may be important indicators of original dietary patterns, including age, ethnicity, place of residence (rural/urban), education, and wealth, in order to reduce the potential confounding effect of diet on the observed associations. The temporal relationships for other factors are discussed in their respective sections above. We were not able to consider in our analyses the use of medications for relieving GERD symptoms. However, as we considered the most frequent and severe symptoms anytime in life as the frequency and severity of symptoms in respective participants, any alleviation of symptoms following the use of medications is unlikely to have had major effects on the observed associations.

### Conclusions

GERD is common in Golestan Province. Several factors associated with GERD in other populations were associated with GERD in our study as well. We also observed associations of hookah and opium use and an inverse association of nass use with GERD. These associations, like many other currently known ones, may not be causal and merit further investigation. Several modifiable lifestyle factors have consistently been associated with GERD. The possibility that modifying these factors may alleviate or prevent GERD symptoms needs to be clarified in controlled studies.

## Supporting Information

Table S1 **Frequency and severity of gastroesophageal reflux disease (GERD) symptoms in two time periods (the last year before interview and earlier).**
(DOCX)

Table S2 **Association of sociodemographic and lifestyle factors with <weekly and weekly GERD symptoms.**
(DOCX)

Table S3 **Association of sociodemographic and lifestyle factors with mild and moderate GERD symptoms.**
(DOCX)

Table S4 **Association between several demographic and lifestyle factors and first start of gastroesophageal reflux symptoms (≥weekly).**
(DOCX)
