# Supplementary material for: Determinants of Gastroesophageal Reflux Disease, Including Hookah Smoking and Opium Use– A Cross-Sectional Analysis of 50,000 Individuals
Source: PLoS One. 2014 Feb 21;9(2):e89256. doi: 10.1371/journal.pone.0089256 (PMC3931722; doi:10.1371/journal.pone.0089256)
Supplement: Table S1 — Frequency and severity of gastroesophageal reflux disease (GERD) symptoms in two time periods (the last year before interview and earlier). (DOCX) [file pone.0089256.s001.docx]

**Table S1.** Frequency and severity of gastroesophageal reflux disease (GERD) symptoms in two time periods (the last year before interview and earlier)

| **Frequency** | ***Last year*** |  |  |  |  |
| --- | --- | --- | --- | --- | --- |
| ***Before last year*** | Never | <weekly | Weekly | Daily | Total |
| Never | 19,560 (79.94) | 3139 (12.83) | 798 (3.26) | 972 (3.97) | 24,469 (100) |
| <weekly | 737 (4.09) | 16,595 (92.09) | 360 (2.00) | 328 (1.82) | 18,020 (100) |
| Weekly | 123 (3.80) | 193 (5.96) | 2555 (78.96) | 365 (11.28) | 3236 (100) |
| Daily | 301 (7.08) | 284 (6.68) | 105 (2.47) | 3560 (83.76) | 4250 (100) |
| Total | 20,721 (41.46) | 20,211 (40.44) | 3818 (7.64) | 5225 (10.46) | 49,975 (100) |
| **Severity** | ***Last year*** |  |  |  |  |
| ***Before last year*** | Never | Mild | Moderate | Severe | Total |
| Never | 19,558 (79.88) | 1199 (4.89) | 3005 (12.27) | 725 (2.96) | 24,487 (100) |
| Mild | 164 (4.50) | 3086 (84.64) | 357 (9.79) | 39 (1.07) | 3646 (100) |
| Moderate | 774 (4.38) | 342 (1.93) | 15,837 (89.45) | 751 (4.24) | 17704 (100) |
| Severe | 232 (5.59) | 55 (1.33) | 388 (9.35) | 3473 (83.73) | 4148 (100) |
| Total | 20,728 (41.49) | 4682 (9.36) | 19,587 (39.18) | 4988 (9.98) | 49,985 (100) |

The values are numbers (percentages). The weighted kappa statistic for the agreement between the two time periods was 0.75 for frequency and 0.76 for severity of GERD symptoms.
