# Supplementary material for: Determinants of Gastroesophageal Reflux Disease, Including Hookah Smoking and Opium Use– A Cross-Sectional Analysis of 50,000 Individuals
Source: PLoS One. 2014 Feb 21;9(2):e89256. doi: 10.1371/journal.pone.0089256 (PMC3931722; doi:10.1371/journal.pone.0089256)
Supplement: Table S2 — Association of sociodemographic and lifestyle factors with <weekly and weekly GERD symptoms. (DOCX) [file pone.0089256.s002.docx]

**Table S2.** Association of sociodemographic and lifestyle factors with <weekly and weekly GERD symptoms

| **Variables** | **<Weekly** |  | **Weekly** |  |
| --- | --- | --- | --- | --- |
|  | N (%) | OR (95% CI) | N (%) | OR (95% CI) |
| **Total** | 20,471 (100) | – | 4029 | – |
| **Age** * | 52.0 (8.9) | 1.02 (1.00-1.05) | 52.1 (9.1) | 0.98 (0.94-1.02) |
| **Sex** |  |  |  |  |
| Women | 11,983 (58.54) | Referent | 2603 (64.61) | Referent |
| Men | 8488 (41.46) | 0.61 (0.58-0.65) | 1426 (35.39) | 0.51 (0.46-0.56) |
| **Ethnicity** |  |  |  |  |
| Non-Turkmen | 4548 (22.22) | Referent | 1318(32.71) | Referent |
| Turkmen | 15923 (77.78) | 1.27 (1.21-1.34) | 2711 (67.29) | 0.75 (0.69-0.82) |
| **Residence** |  |  |  |  |
| Rural | 15,549 (75.96) | Referent | 3031 (75.23) | Referent |
| Urban | 4921 (24.04) | 1.39 (1.31-1.47) | 998 (24.77) | 1.46 (1.33-1.61) |
| **Education** |  |  |  |  |
| No school | 14,086 (68.81) | Referent | 2994 (74.31) | Referent |
| 1 – 8^th^ grade | 4454 (21.76) | 1.02 (0.97-1.08) | 773 (19.19) | 0.89 (0.80-0.98) |
| High School | 1426 (6.97) | 1.05 (0.95-1.15) | 184 (4.57) | 0.74 (0.62-0.89) |
| Higher | 505 (2.47) | 1.22 (1.05-1.41) | 78 (1.94) | 1.12 (0.86-1.47) |
| *P* for trend |  | 0.03 |  | 0.04 |
| **Wealth score** |  |  |  |  |
| Quintile 1-lowest | 5106 (24.94) | Referent | 1308 (32.70) | Referent |
| Quintile 2 | 3457 (16.89) | 1.03 (0.96-1.09) | 635 (32.46) | 0.81 (0.73-0.90) |
| Quintile 3 | 3999 (19.53) | 0.98 (0.92-1.04) | 760 (18.86) | 0.81 (0.73-0.90) |
| Quintile 4 | 3445 (16.83) | 0.95 (0.89-1.01) | 619 (15.36) | 0.74 (0.66-0.82) |
| Quintile 5 | 4464 (21.81) | 0.98 (0.92-1.05) | 707 (17.55) | 0.67 (0.60-0.76) |
| *P* for trend |  | 0.17 |  | <0.001 |
| **Body mass index** |  |  |  |  |
| <18.5 kg/m^2^ | 854 (4.17) | 0.88 (0.80-0.97) | 240 (5.96) | 1.08 (0.93-1.26) |
| 18.5 – 24.9 | 6944 (33.93) | Referent | 1425 (35.37) | Referent |
| 25 – 29.9 | 7148 (34.92) | 1.14 (1.09-1.20) | 1281 (31.79) | 1.05 (0.96-1.14) |
| ≥30 | 5522 (26.98) | 1.20 (1.13-1.26) | 1083 (26.88) | 1.17 (1.07-1.29) |
| *P* for trend |  | <0.001 |  | 0.01 |
| **Physical activity** |  |  |  |  |
| Irregular non-intense | 12,184 (59.8) | Referent | 2606 (64.87) | Referent |
| Regular non-intense | 6034 (29.6) | 1.17 (1.11-1.23) | 984 (24.50) | 1.05 (0.96-1.15) |
| Regular or irregular intense | 2172 (10.6) | 1.08 (1.01-1.16) | 427 (10.63) | 0.99 (0.87-1.12) |
| *P* for trend |  | <0.001 |  | 0.85 |
| **Alcohol drinking** |  |  |  |  |
| Never | 19,724 (96.35) | Referent | 3869 (96.03) | Referent |
| Ever | 747 (3.65) | 0.98 (0.87-1.10) | 160 (3.97) | 1.39 (1.14-1.69) |
| **Cigarette smoking** |  |  |  |  |
| Never | 16,783 (82.02) | Referent | 3355 (83.31) | Referent |
| 0.1 – 5 pack-years | 1167 (5.7) | 1.21 (1.10-1.32) | 207 (5.14) | 1.15 (0.98-1.36) |
| 5.1 – 10 | 539 (2.63) | 1.28 (1.12-1.46) | 109 (2.71) | 1.43 (1.14-1.79) |
| 10.1 – 20 | 808 (3.95) | 1.36 (1.22-1.52) | 144 (3.58) | 1.28 (1.05-1.57) |
| ≥20 | 1166 (5.70) | 1.28 (1.16-1.41) | 212 (5.26) | 1.19 (1.00-1.42) |
| *P* for trend |  | <0.001 |  | 0.003 |
| **Hookah smoking** |  |  |  |  |
| Never | 20,264 (99.04) | Referent | 3964 (98.44) | Referent |
| Ever | 197 (0.96) | 1.18 (0.96-1.45) | 63 (1.56) | 1.25 (0.93-1.68) |
| **Nass chewing** |  |  |  |  |
| Never | 18,969 (92.68) | Referent | 3714 (92.18) | Referent |
| Ever | 1499 (7.32) | 1.00 (0.92-1.08) | 315 (7.82) | 1.02 (0.88-1.18) |
| **Opium use** |  |  |  |  |
| Never | 16,958 (82.85) | Referent | 3250 (80.69) | Referent |
| Ever | 3510 (17.15) | 1.30 (1.22-1.39) | 778 (19.31) | 1.56 (1.40-1.73) |

Numbers may not add up to the total numbers due to missing data. The ORs (95% CIs) were calculated using multinomial logistic regression models, in which <weekly, weekly, and daily symptoms, as separate categories were compared with never having GERD symptoms. Results for daily symptoms are shown in Table 2. The ORs (95% CIs) are from multivariate models in which all the variables shown in this table were included.

* For age, the values are mean (standard deviation) years. Age was included in the models as a continuous variable, but the ORs (95% CIs) are shown here on a 10-year scale.
