# Supplementary material for: Determinants of Gastroesophageal Reflux Disease, Including Hookah Smoking and Opium Use– A Cross-Sectional Analysis of 50,000 Individuals
Source: PLoS One. 2014 Feb 21;9(2):e89256. doi: 10.1371/journal.pone.0089256 (PMC3931722; doi:10.1371/journal.pone.0089256)
Supplement: Table S3 — Association of sociodemographic and lifestyle factors with mild and moderate GERD symptoms. (DOCX) [file pone.0089256.s003.docx]

**Table S3.** Association of sociodemographic and lifestyle factors with mild and moderate GERD symptoms

| **Variables** | **Mild** |  | **Moderate** |  |
| --- | --- | --- | --- | --- |
|  | N (%) | OR (95% CI) | N (%) | OR (95% CI) |
| **Total** | 4449 (100) | – | 20,315 (100) | – |
| **Age** * | 51.9 (9.0) | 1.00 (0.96-1.04) | 52.2 (9.0) | 1.03 (1.01-1.06) |
| **Sex** |  |  |  |  |
| Women | 2368 (53.23) | Referent | 12,481 (61.44) | Referent |
| Men | 2081 (46.77) | 0.76 (0.70-0.84) | 7834 (38.56) | 0.56 (0.52-0.59) |
| **Ethnicity** |  |  |  |  |
| Non-Turkmen | 1378 (30.97) | Referent | 5015 (24.69) | Referent |
| Turkmen | 3071 (69.03) | 0.89 (0.82-0.96) | 15,300 (75.31) | 1.08 (1.03-1.13) |
| **Residence** |  |  |  |  |
| Rural | 3000 (67.45) | Referent | 15,874 (78.14) | Referent |
| Urban | 1448 (32.55) | 1.77 (1.62-1.93) | 4441 (21.86) | 1.26 (1.18-1.33) |
| **Education** |  |  |  |  |
| No school | 2744 (61.68) | Referent | 14,573 (71.74) | Referent |
| 1 – 8^th^ grade | 1128 (25.35) | 1.09 (0.99-1.19) | 4168 (20.52) | 1.00 (0.94-1.06) |
| High School | 425 (9.55) | 1.19 (1.03-1.37) | 1162 (5.72) | 0.98 (0.87-1.07) |
| Higher | 152 (3.42) | 1.30 (1.05-1.61) | 412 (2.03) | 1.19 (1.02-1.38) |
| *P* for trend |  | 0.003 |  | 0.38 |
| **Wealth score** |  |  |  |  |
| Quintile 1-lowest | 1092 (24.54) | Referent | 5384 (26.5) | Referent |
| Quintile 2 | 581 (13.06) | 0.85 (0.76-0.95) | 3554 (17.49) | 1.03 (0.97-1.09) |
| Quintile 3 | 828 (18.61) | 0.96 (0.86-1.06) | 4001 (19.69) | 0.97 (0.91-1.03) |
| Quintile 4 | 770 (17.31) | 0.93 (0.84-1.03) | 3430 (16.88) | 0.95 (0.89-1.01) |
| Quintile 5 | 1178 (26.48) | 1.02 (0.92-1.14) | 3946 (19.42) | 0.90 (0.84-0.96) |
| *P* for trend |  | 0.64 |  | 0.001 |
| **Body mass index** |  |  |  |  |
| <18.5 kg/m^2^ | 170 (3.82) | 0.85 (0.72-1.01) | 952 (4.69) | 0.93 (0.84-1.02) |
| 18.5 – 24.9 | 1518 (34.13) | Referent | 6986 (34.39) | Referent |
| 25 – 29.9 | 1585 (35.63) | 1.11 (1.02-1.20) | 6952 (34.22) | 1.13 (1.08-1.19) |
| ≥30 | 1175 (26.42) | 1.13 (1.03-1.24) | 5423 (26.7) | 1.19 (1.12-1.25) |
| *P* for trend |  | <0.001 |  | <0.001 |
| **Physical activity** |  |  |  |  |
| Irregular non-intense | 2397 (54.23) | Referent | 12,882 (63.64) | Referent |
| Regular non-intense | 1490 (33.71) | 1.22 (1.12-1.33) | 5205 (25.71) | 1.04 (0.99-1.10) |
| Regular or irregular intense | 533 (12.06) | 1.17 (1.04-1.32) | 2155 (10.65) | 1.03 (0.96-1.11) |
| *P* for trend |  | <0.001 |  | 0.25 |
| **Alcohol drinking** |  |  |  |  |
| Never | 4222 (94.9) | Referent | 19,661 (96.78) | Referent |
| Ever | 227 (5.1) | 1.18 (0.99-1.40) | 654 (3.22) | 0.97 (0.86-1.09) |
| **Cigarette smoking** |  |  |  |  |
| Never | 3608 (81.17) | Referent | 16,797 (82.71) | Referent |
| 0.1 – 5 pack-years | 272 (6.12) | 1.16 (1.00-1.34) | 1108 (5.46) | 1.21 (1.10-1.33) |
| 5.1 – 10 | 126 (2.83) | 1.21 (0.98-1.49) | 519 (2.56) | 1.32 (1.15-1.50) |
| 10.1 – 20 | 166 (3.73) | 1.10 (0.91-1.33) | 764 (3.76) | 1.37 (1.22-1.54) |
| ≥20 | 273 (6.14) | 1.21 (1.03-1.41) | 1121 (5.52) | 1.28 (1.16-1.41) |
| *P* for trend |  | 0.01 |  | <0.001 |
| **Hookah smoking** |  |  |  |  |
| Never | 4390 (98.67) | Referent | 20,084 (98.92) | Referent |
| Ever | 59 (1.33) | 1.31 (0.97-1.77) | 219 (1.08) | 1.15 (0.94-1.41) |
| **Nass chewing** |  |  |  |  |
| Never | 4138 (93.01) | Referent | 18,783 (92.47) | Referent |
| Ever | 311 (6.99) | 1.01 (0.88-1.16) | 1529 (7.53) | 1.00 (0.92-1.09) |
| **Opium use** |  |  |  |  |
| Never | 3773 (84.88) | Referent | 16,609 (81.76) | Referent |
| Ever | 672 (15.12) | 1.10 (0.98-1.22) | 3706 (18.24) | 1.43 (1.34-1.52) |

Numbers may not add up to the total numbers due to missing data. The ORs (95% CIs) were calculated using multinomial logistic regression models, in which mild, moderate, and severe symptoms, as separate categories were compared with never having GERD symptoms. Results for severe symptoms are shown in Table 2. The ORs (95% CIs) are from multivariate models in which all the variables shown in this table were included. The severity of symptoms here was defined as: mild, the study participant did not feel the symptoms unless they actively paid attention; moderate, the study participant felt the symptoms without active attention, but they did not interfere with daily work.

* For age, the values are mean (standard deviation) years. Age was included in the models as a continuous variable, but the ORs (95% CIs) are shown here on a 10-year scale.
