# Supplementary material for: Determinants of Gastroesophageal Reflux Disease, Including Hookah Smoking and Opium Use– A Cross-Sectional Analysis of 50,000 Individuals
Source: PLoS One. 2014 Feb 21;9(2):e89256. doi: 10.1371/journal.pone.0089256 (PMC3931722; doi:10.1371/journal.pone.0089256)
Supplement: Table S4 — Association between several demographic and lifestyle factors and first start of gastroesophageal reflux symptoms (≥weekly). (DOCX) [file pone.0089256.s004.docx]

**Table S4.** Association between several demographic and lifestyle factors and first start of gastroesophageal reflux symptoms (≥weekly)

|  | **Symptom start** |  |  |  |
| --- | --- | --- | --- | --- |
| **Variables** | **<1 year ago** | **1 – 5 years ago** | **6 – 10 years ago** | **>10 years ago** |
| **Age** * | 0.98 (0.92-1.04) | 0.98 (0.94-1.02) | 1.00 (0.94-1.06) | 1.21 (1.15-1.27) |
| **Sex** |  |  |  |  |
| Women | Referent | Referent | Referent | Referent |
| Men | 0.37 (0.32-0.43) | 0.43 (0.39-0.48) | 0.43 (0.36-0.50) | 0.38 (0.33-0.43) |
| **Turkmen** |  |  |  |  |
| Non-Turkmen | Referent | Referent | Referent | Referent |
| Turkmen | 0.56 (0.50-0.63) | 0.65 (0.60-0.70) | 0.84 (0.74-0.95) | 0.82 (0.74-0.90) |
| **Residence** |  |  |  |  |
| Rural | Referent | Referent | Referent | Referent |
| Urban | 1.02 (0.88-1.18) | 1.07 (0.97-1.19) | 1.15 (0.99-1.34) | 1.48 (1.32-1.65) |
| **Education** |  |  |  |  |
| No school | Referent | Referent | Referent | Referent |
| 1 – 8^th^ grade | 0.69 (0.59-0.81) | 0.83 (0.75-0.93) | 0.94 (0.80-1.10) | 1.20 (1.07-1.35) |
| High School | 0.55 (0.40-0.77) | 0.71 (0.58-0.87) | 0.83 (0.62-1.10) | 1.01 (0.81-1.24) |
| Higher | 0.50 (0.26-0.96) | 0.76 (0.53-1.09) | 1.03 (0.65-1.63) | 1.77 (1.33-2.37) |
| **Wealth score** |  |  |  |  |
| Quintile 1 | Referent | Referent | Referent | Referent |
| Quintile 2 | 0.82 (0.71-0.94) | 0.85 (0.76-0.94) | 0.85 (0.73-1.00) | 0.82 (0.72-0.94) |
| Quintile 3 | 0.82 (0.71-0.94) | 0.81 (0.73-0.90) | 0.84 (0.72-0.99) | 1.01 (0.90-1.15) |
| Quintile 4 | 0.63 (0.54-0.74) | 0.72 (0.64-0.80) | 0.89 (0.76-1.05) | 0.96 (0.84-1.09) |
| Quintile 5 | 0.53 (0.44-0.63) | 0.56 (0.50-0.64) | 0.77 (0.65-0.92) | 0.91 (0.80-1.04) |
| **Body mass index** |  |  |  |  |
| <18.5 kg/m2 | 1.10 (0.89-1.36) | 0.91 (0.77-1.08) | 1.02 (0.80-1.30) | 1.10 (0.91-1.32) |
| 18.5 – 24.9 | Referent | Referent | Referent | Referent |
| 25 – 29.9 | 1.12 (0.99-1.26) | 1.12 (1.02-1.22) | 1.06 (0.93-1.21) | 1.06 (0.96-1.18) |
| ≥30 | 1.09 (0.95-1.25) | 1.22 (1.11-1.35) | 1.20 (1.05-1.38) | 1.16 (1.04-1.30) |
| **Physical activity** |  |  |  |  |
| Irregular non-intense | Referent | Referent | Referent | Referent |
| Regular non-intense | 0.98 (0.85-1.14) | 0.96 (0.87-1.06) | 0.90 (0.78-1.05) | 1.03 (0.92-1.15) |
| Regular or irregular intense | 1.02 (0.86-1.21) | 0.96 (0.85-1.09) | 1.02 (0.84-1.23) | 0.85 (0.73-1.00) |
| **Alcohol drinking** |  |  |  |  |
| Never | Referent | Referent | Referent | Referent |
| Ever | 1.55 (1.11-2.17) | 1.52 (1.23-1.88) | 1.14 (0.82-1.59) | 1.21 (0.96-1.52) |
| **Cigarette smoking** |  |  |  |  |
| Never | Referent | Referent | Referent | Referent |
| 0.1 – 5 pack-years | 0.96 (0.74-1.26) | 1.21 (1.01-1.44) | 1.23 (0.95-1.59) | 1.26 (1.03-1.53) |
| 5.1 – 10 | 1.34 (0.93-1.92) | 1.39 (1.08-1.78) | 1.42 (0.99-2.04) | 1.36 (1.03-1.80) |
| 10.1 – 20 | 1.07 (0.76-1.51) | 1.27 (1.02-1.58) | 1.26 (0.90-1.74) | 1.42 (1.11-1.80) |
| ≥20 | 1.06 (0.80-1.41) | 1.35 (1.12-1.62) | 1.31 (0.99-1.72) | 1.50 (1.23-1.84) |
| **Hookah smoking** |  |  |  |  |
| Never | Referent | Referent | Referent | Referent |
| Ever | 1.26 (0.87-1.83) | 1.11 (0.82-1.51) | 1.19 (0.75-1.88) | 1.20 (0.84-1.70) |
| **Nass chewing** |  |  |  |  |
| Never | Referent | Referent | Referent | Referent |
| Ever | 1.15 (0.94-1.42) | 0.96 (0.83-1.12) | 0.93 (0.73-1.17) | 0.79 (0.66-0.96) |
| **Opium use** |  |  |  |  |
| Never | Referent | Referent | Referent | Referent |
| Ever | 1.56 (1.34-1.82) | 1.72 (1.54-1.91) | 1.64 (1.40-1.92) | 1.90 (1.68-2.14) |

The ORs (95% CIs) are from multivariate, multinomial logistic regression models in which the above categories of symptom onset were compared with never having GERD symptoms and all sociodemographic and lifestyle factors shown in this table were included.

* Age was included in the models as a continuous variable, but the ORs (95% CIs) are shown here on a 10-year scale.
